# Editorial special issue: Statistics in sports

**DOI:** 10.1007/s10182-022-00453-9

**Published:** 2022-07-11

**Authors:** Andreas Groll, Dominik Liebl

**Affiliations:** 1 Fakultät Statistik, TU Dortmund University, Vogelpothsweg 87, 44221 Dortmund, Germany; 2 Institute of Finance and Statistics, University Bonn, Adenauerallee 24-26, 53113 Bonn, Germany

## Abstract

Triggered by advances in data gathering technologies, the use of statistical analyzes, predictions and modeling techniques in sports has gained a rapidly growing interest over the last decades. Today, professional sports teams have access to precise player positioning data and sports scientists design experiments involving non-standard data structures like movement-trajectories. This special issue on statistics in sports is dedicated to further foster the development of statistics and its applications in sports. The contributed articles address a wide range of statistical problems such as statistical methods for prediction of game outcomes, for prevention of sports injuries, for analyzing sports science data from movement laboratories, for measurement and evaluation of player performance, etc. Finally, also SARS-CoV-2 pandemic-related impacts on the sport’s framework are investigated.

## Football

Ötting and Mews (2022) investigate on German Bundesliga data the potential occurrence of change points—commonly referred to as “momentum shifts”—in the dynamics of football matches. For that purpose, minute-by-minute in-game match statistics are modeled via hidden Markov models. To allow for within-state correlation of the variables considered, multivariate state-dependent distributions using copulas are formulated. The fitted HMMs indeed comprise states which can be interpreted as a team showing different levels of control over a match.

Dick and Brefeld (2022) present a data-driven approach to predict the next action in soccer, with focus on passing actions of the ball-possessing player. The aim is to forecast the pass itself and when, in time, it will be played. Simultaneously, the model estimates the probability that the player loses possession of the ball before she can perform the action. The approach consists of parameterized exponential rate models for all possible actions that are adapted to historic data with graph recurrent neural networks to account for inter-dependencies of the output space (i.e., the possible actions).

Fadel (2022) consider probabilistic movement models that allow predicting players’ positions over time and are thus valuable modeling approaches in sports analytic problems. The authors propose a novel sport analytics approach based on normalizing flows for estimating the conditional densities of the movement model given arbitrary contexts (e.g., running speeds, etc.). The novel modeling approach is compared with several benchmark approaches using professional soccer data from the German football league (Bundesliga).

Porro and Zenga (2022) extend existing results on the Pietra index, a measure for inequality similar to but different from the Gini-Index. The authors propose novel decompositions by sources and by sub-populations. These novel inequality measures are applied successfully to measuring inequalities in balance sheet data of professional Italian soccer teams (Serie A) and inequalities in the market values of Serie A (Italy) soccer players.

Zumeta-Olaskoaga (2022) consider the case of predicting sports injuries. Major challenges in predicting sports injuries are often (i) small sample sizes, (ii) even fewer number of injuries, but (iii) large numbers of potential predictor variables. To overcome these challenges, the authors propose a shared frailty Cox model to predict the occurrence of sports injuries in football-focusing on lower-limb injuries that frequently occur in women football. Different variable selection methods (best subset regression, lasso, ridge, elastic net, and group lasso) are assessed with respect using a real-data case study and simulation studies.

van der Wurp and Groll (2022) propose a bivariate Poisson modeling approach to take into account the dependencies of bivariate outcome variables, such as the number of goals of the home and guest team in soccer. To allow for shrinkage of regression coefficient estimates and variable selection, the authors propose to extend the generalized joint regression modeling (GJRM) infrastructure of Marra and Radice (2020) by adding an (adaptive) lasso-type penalization based on a quadratic lasso approximation originally proposed in Oelker and Tutz (2017). The performance of the novel modeling approach is assessed in a simulation study and a case study application on FIFA World Cup data football data.

Ievoli et al. (2022) use Bayesian hierarchical models to assess the role of passing network indicators for match outcome predictions. Passing networks can characterize the differences of two competing soccer teams and thus may serve as predictors for predicting match outcomes. Bayesian hierarchical models with novel network-based predictors and more classic in-match predictors are proposed. The usefulness of network-based predictors is demonstrated in a real data study based on UEFA Champions League data. Passing speed, passing balance, and the teams cohesion in terms of passing behavior can be valuable network-based predictors for football game outcomes.

Hanck and Arnold (2022) propose a hierarchical Bayes model to rank soccer penalty-takers in the German Bundesliga based on historical data from 1963 to 2021. A problem in such data is that many players are only rarely penalty-takers, and thus, their relative goal frequency may be a bad estimate of their actual conversion rate. To solve this problem (among others), a shrinkage estimation approach is proposed using Bayesian beta-binomial models, which allows borrowing information from players who have taken many penalties. The authors demonstrate that such a hierarchical Bayesian model that allows to model the accuracy of all players can have advantages over a purely frequentist approach.

## Football-COVID

Benz and Lopez (2022) estimate the change in soccer’s home advantage during the Covid-19 pandemic using bivariate Poisson regression. The ghost games that took place in the postponed football matches across the world in spring 2020 due to the COVID-19 pandemic allowed researchers from many disciplines to compare the rescheduled games, played in front of empty stadia, to previous games, played in front of fans. To date, most of this post-COVID football research has used linear regression models, or versions thereof, to estimate potential changes to the home advantage. However, it is shown here that leveraging the Poisson distribution is more appropriate. Particularly, in simulations, it is shown that bivariate Poisson regression (Karlis and Ntzoufras 2003) reduces absolute bias when estimating the home advantage benefit in a single season of football games, relative to linear regression, by almost 85 percent. Moreover, with data from 17 professional football leagues, the bivariate Poisson models are then extended to estimate the change in home advantage due to games being played without fans.

Gorgi (2022) propose a novel approach to determine the final league table in football competitions with a premature ending. As for several countries, a premature ending of the 2019/2020 football season has occurred due to the COVID-19 pandemic, and a model-based method is proposed as a possible alternative to the use of the incomplete standings to determine the final table. This method measures the performance of the teams in the matches of the season that have been played and predicts the remaining non-played matches through a paired-comparison model. The main advantage of the method compared to the incomplete standings is that it takes account of the bias in the performance measure due to the schedule of the matches in a season and hence can be regarded as fairer in this respect.

Van Eetvelde et al. (2022) also consider the problem of determining the final standing in abruptly stopped football seasons. Similar to Gorgi (2022), a statistical solution is proposed to predict a fair final standing in abruptly stopped football seasons. By contrast to Gorgi (2022), the authors consider a simulation-based approach that computes the probabilities for each possibly final rank by simulating outcomes overall non-played matches. This leads to a probabilistic final standing that is much richer in terms of information than simply the expected final standing. The approach is evaluated using data from the top soccer leagues of England, France, Germany, Italy, and Spain, for the 2016–2017, 2017–2018 and 2018–2019 seasons.

## Basketball

Migliorati et al. (2022) investigate the importance of Oliver’s Four Factors, which are used to identify a team’s strengths and weaknesses in terms of shooting, turnovers, rebounding and free throws, on success in basketball. The role of each factor in the success of a team in a match is analyzed via the MOdel-Based recursive partitioning (MOB) algorithm on data covering 19138 matches of 16 National Basketball Association regular seasons (from 2004–2005 to 2019–2020). MOB, instead of fitting one global generalized linear model (GLM) to all observations, partitions the observations according to selected partitioning variables and estimates several ad hoc local GLMs for subgroups of observations.

Ekstrom and Jensen (2022) evaluate scoring streaks and game excitement using in-match trend estimation. While for many popular sports, mostly, the overall match winner and result are interesting, it often conveys little information about the underlying scoring trends throughout the match. Modeling approaches that accommodate a finer granularity of the score difference throughout the match are needed to evaluate in-game strategies, discuss scoring streaks, teams’ strengths, and other aspects of the game. Here, a latent Gaussian process to model the score difference between two teams is proposed, and the Trend Direction Index is introduced as an easily interpretable probabilistic measure of the current trend in the match as well as a measure of post-game trend evaluation. In addition, the Excitement Trend Index—the expected number of monotonicity changes in the running score difference—is proposed as a measure of overall game excitement. The proposed methods are applied to all matches from the 2019 to 2020 NBA basketball season.

Mews and Ötting (2022) investigate the hot hand hypothesis in basketball. The hot hand hypothesis postulates that athletes may temporally enter a state during which they stably perform better than on average. The authors propose to analyze this hypothesis using a state-space modeling approach that allows for time periods with increased success probabilities. The hidden (unobserved) player’s form is modeled using a stationary stochastic process, which allows for a continuum of players forms-rather than a finite set of discrete states. By contrast to many existing approaches, this modeling approach directly allows for time series data with unevenly spaced time points. For estimation, the continuous state-space is discretized which allows approximate inference using a continuous-time hidden Markov model (HMM) with a large but finite number of states. Using this model, the authors provide evidence for the existence of a (small) hot hand effect in basketball free throws using data from the National Basketball Association (NBA).

## NFL

Reyers and Swartz (2022) propose a novel procedure to evaluate quarterbacks in the National Football League (NFL). Today, quantitative performance evaluation procedures use rich tracking data to assess the expected points gained from various options that are available to quarterbacks in the NFL. However, measuring the performance of quarterbacks is a highly complex problem since the evaluation of quarterbacks ideally should not depend on the quality of his team, even though the interactions with his team are evaluated. The authors attempt to introduce a novel quarterback metric which is less dependent on the performance of one’s teammates than other existing performance evaluation procedures in the literature. The idea is to compare a quarterback’s outcomes with the performance of the hypothetical quarterback, who makes optimal decisions. That is, a quarterback receives high-performance scores if he uses the available options most efficiently. The authors propose a machine learning approach (ensemble of base learners) to estimate the expected points of the available options and their successful execution probabilities. The usefulness of the novel method is demonstrated using historical NFL data.

## Swimming

Fabbricatore et al. (2022) propose a component-based structural equation modeling approach for the assessment of psycho-social aspects and performance of athletes. Performance in elite sports also depends on the personality traits of the athletes. Using a component-based structural equation model based on a partial least squares estimation approach, the authors investigate (a) which personality traits mostly affect swimmers’ mental skills and (b) the effect of mental skills on their performance. A sample of 161 young elite swimmers enrolled in the Italian Swimming Federation (Campania Regional Committee) is considered. The findings of this study will be useful for designing novel strategies and interventions for coaches as well as swimmers to maximize the performance and well-being.

## Sports biomechanics

Pataky et al. (2022) focus on the inferential problem of identifying the parts of a functional domain where two population means differ. Four approaches recently used in sports science are considered: interval-wise testing, statistical parametric mapping, statistical nonparametric mapping and the Benjamini-Hochberg procedure for false discovery control. These procedures are applied to both six representative sports science datasets, and also to systematically varied simulated datasets which replicated ten signal- and/or noise-relevant parameters that were identified in the experimental datasets.

## Conclusion

Professional sports and sports science are becoming more and more engaged with statistics and data analysis due to the tremendous progress in data gathering technologies both on the pitch and in the lab. Today, the collected data are often highly complex and may involve network structures like passing networks, and time-continuous movement processes recorded by high-frequency camera systems, etc. This broad range of novel data problems motivates a wide range of novel statistical procedures, which is reflected in this special issue covering statistical tools such as machine learning algorithms (Dick and Brefeld 2022; Fadel 2022), Cox frailty models (Zumeta-Olaskoaga 2022), hidden Markov models (Ötting and Mews 2022; Mews and Ötting 2022), Bayesian hierarchical models (Hanck and Arnold 2022; Ievoli et al. 2022), structural equation modeling (Fabbricatore et al. 2022), random process theory and functional data analysis (Pataky et al. 2022), bivariate Poisson regression (van der Wurp and Groll 2022; Benz and Lopez 2022), and latent Gaussian processes (Ekstrom and Jensen 2022). While all these works address specific statistical problems in sports, this editorial aims to stimulate statistical research with an application to sports in general, and to help non-statisticians understand why and how statistics can be a very valuable tool in this context.
